# Barth syndrome

**DOI:** 10.1186/1750-1172-8-23

**Published:** 2013-02-12

**Authors:** Sarah LN Clarke, Ann Bowron, Iris L Gonzalez, Sarah J Groves, Ruth Newbury-Ecob, Nicol Clayton, Robin P Martin, Beverly Tsai-Goodman, Vanessa Garratt, Michael Ashworth, Valerie M Bowen, Katherine R McCurdy, Michaela K Damin, Carolyn T Spencer, Matthew J Toth, Richard I Kelley, Colin G Steward

**Affiliations:** 1 Department of Paediatrics, Leicester Royal Infirmary, Infirmary Square, Leicester, LE1 5WW, UK; 2 NHS Specialised Services Barth Syndrome Service, Royal Hospital for Children, Upper Maudlin St, Bristol, BS2 8BJ, UK; 3 Molecular Diagnostics Laboratory, Nemours Biomedical Research, Alfred I. duPont Hospital for Children, Wilmington, Delaware, 19899, USA; 4 School of Cellular & Molecular Medicine, Faculty of Medical Sciences, University of Bristol, University Walk, Bristol, BS8 1TD, UK; 5 Department of Histopathology, Great Ormond Street Hospital, Great Ormond Street, London, WC1N 3JH, UK; 6 Barth Syndrome Foundation, Inc., P.O. Box 618, Larchmont, NY, 10538, USA; 7 Barth Syndrome Trust, 1, The Vikings, Romsey, SO51 5RG, UK; 8 Dept of Pediatrics, Division of Cardiology, Medical University of South Carolina, Charleston, SC, USA; 9 Department of Pediatrics, John Hopkins University School of Medicine, Baltimore, MD, USA; 10 Department of Metabolism, Kennedy Krieger Institute, Baltimore, MD, USA; 11 Oncology Day Beds, Royal Hospital for Children, Upper Maudlin St., Bristol, BS2 8BJ, UK

**Keywords:** Barth syndrome, 3-methylglutaconic aciduria, Dilated cardiomyopathy, Stillbirth, Growth delay, Endocardial fibroelastosis, Left ventricular non-compaction, Arrhythmia, Myopathy, Neutropenia

## Abstract

First described in 1983, Barth syndrome (BTHS) is widely regarded as a rare X-linked genetic disease characterised by cardiomyopathy (CM), skeletal myopathy, growth delay, neutropenia and increased urinary excretion of 3-methylglutaconic acid (3-MGCA). Fewer than 200 living males are known worldwide, but evidence is accumulating that the disorder is substantially under-diagnosed. Clinical features include variable combinations of the following wide spectrum: dilated cardiomyopathy (DCM), hypertrophic cardiomyopathy (HCM), endocardial fibroelastosis (EFE), left ventricular non-compaction (LVNC), ventricular arrhythmia, sudden cardiac death, prolonged QTc interval, delayed motor milestones, proximal myopathy, lethargy and fatigue, neutropenia (absent to severe; persistent, intermittent or perfectly cyclical), compensatory monocytosis, recurrent bacterial infection, hypoglycaemia, lactic acidosis, growth and pubertal delay, feeding problems, failure to thrive, episodic diarrhoea, characteristic facies, and X-linked family history. Historically regarded as a cardiac disease, BTHS is now considered a multi-system disorder which may be first seen by many different specialists or generalists. Phenotypic breadth and variability present a major challenge to the diagnostician: some children with BTHS have never been neutropenic, whereas others lack increased 3-MGCA and a minority has occult or absent CM. Furthermore, BTHS was first described in 2010 as an unrecognised cause of fetal death. Disabling mutations or deletions of the *tafazzin* (*TAZ*) gene, located at Xq28, cause the disorder by reducing remodeling of cardiolipin, a principal phospholipid of the inner mitochondrial membrane. A definitive biochemical test, based on detecting abnormal ratios of different cardiolipin species, was first described in 2008. Key areas of differential diagnosis include metabolic and viral cardiomyopathies, mitochondrial diseases, and many causes of neutropenia and recurrent male miscarriage and stillbirth. Cardiolipin testing and *TAZ* sequencing now provide relatively rapid diagnostic testing, both prospectively and retrospectively, from a range of fresh or stored tissues, blood or neonatal bloodspots. *TAZ* sequencing also allows female carrier detection and antenatal screening. Management of BTHS includes medical therapy of CM, cardiac transplantation (in 14% of patients), antibiotic prophylaxis and granulocyte colony-stimulating factor (G-CSF) therapy. Multidisciplinary teams/clinics are essential for minimising hospital attendances and allowing many more individuals with BTHS to live into adulthood.

## Review

### Disease names/synonyms

– Barth Syndrome (BTHS)

– 3-methylglutaconic aciduria type 2

– X-linked cardioskeletal myopathy and neutropenia

– Cardioskeletal myopathy with neutropenia and abnormal mitochondria

– Endocardial fibroelastosis type 2 (EFE2)

### Definition

Dr Peter Barth first described a triad of cardiomyopathy (CM), skeletal myopathy and neutropenia in 1983 in a large Dutch kindred with high infant mortality due to infection or cardiac failure [1], although the first description of the disorder now widely known as Barth Syndrome (BTHS) may have occurred in 1979 [2]. Kelley *et al* added organic aciduria to the definition and described partial phenotypes (isolated neutropenia and lack of CM in respective cases) [3] and Spencer *et al* noted that 6% of 73 known Barth patients had no evidence of CM [4]. In 2010 BTHS was reported as a cause of male fetal death resulting in miscarriage and stillbirth [5].

BTHS has a unique pathogenesis: it is the only known human disease where the primary defect is remodeling of cardiolipin, a phospholipid found in mitochondrial membranes [6]. The disease affects many body systems from fetal through to adult life, making this an important condition for obstetricians, geneticists, general paediatricians, cardiologists and neurologists to be aware of, especially since rapid definitive biochemical testing has recently become available [7].

### Methods

This review contains some references to previously unpublished data collated by the Barth Syndrome Foundation (BSF) Registry. The BSF Registry was approved by the IRBs of the two relevant host institutions, the University of Florida and Boston Children’s Hospital. Inclusion criteria for the Registry are a diagnosis of BTHS, mutation of the causative gene and the provision of informed consent.

### Epidemiology

151 living BTHS patients are known to the BSF worldwide in 2012. In the USA, approximately 10 new patients are diagnosed each year in the USA and 71 kindreds have been identified, and the BSF has estimated a prevalence of 1/300,000-400,000 live births [8]. There is no known racial or ethnic predilection.

Evidence is, however, growing that the disease may be under-diagnosed. In 1999, Cantlay *et al* reported five unrelated BTHS families identified from South-West England and South Wales over a period of seven years [9]. Further case ascertainment has now identified a total of 13 cases in current generations of 8 unrelated families from this regional population of 6 million, implying a prevalence potentially as high as 1/140,000 live births or 0.22/100,000 people (Orphanet category <1-9/1,000,000). Including the cases already described, the NHS Specialised Services Barth Syndrome Service (NSSBSS) in the UK (2011 population: 62.6 million) has so far identified a total of 30 unrelated families affected by the disease in the UK, of whom 22 boys or men are alive from 17 unrelated families. Further information about frequency is described in the section on cardiological aspects of the disease.

### Aetiology

#### Genetics

The primary gene defect in BTHS is mutation in the *tafazzin* (*TAZ*, previously termed G4.5) gene [10], comprising 11 exons and located on Xq28 [11,12]. The *TAZ* sequence is highly conserved in evolutionary terms [13]. There are already more than 120 different mutations identified (data from the Human Tafazzin (*TAZ*) Gene Mutation and Variation Database [14]). Most are missense mutations and small insertions or deletions, but a minority of patients have large exon, or in one case whole gene, deletion [15]. Frameshift mutations causing tafazzin truncation and mutations affecting splice donor or acceptor sites have also been identified. Mutations have been reported in all exons of TAZ, including a variant of unknown significance in exon 5 [16]. No genotype/phenotype correlations have so far been identified and there may be marked phenotypic variation between males within a family (e.g. those described in [17]).

Data from the Human Tafazzin (*TAZ*) Gene Mutation and Variation Database shows that only 13% of boys carry *de novo* mutations not identified in their mother’s somatic DNA [14]. Gonadal mosaicism has also been documented [18], raising the small possibility that a woman who does not herself carry mutations in her somatic DNA may have more than one affected boy.

Although it is theoretically possible for a female to manifest symptoms of BTHS due to skewed X-inactivation, the only female ever described with the disease had abnormalities of both X chromosomes [19]. One was a ring form with a large deletion of the long arm (including the Xq28 region) and the second had a large deletion of exons 1–5 of *TAZ*. Problems in this patient included intrauterine growth retardation and the development of severe dilated cardiomyopathy (DCM) with left ventricular non-compaction (LVNC) at one month of age.

Among 16 obligate BTHS carriers from six families whose fibroblasts or blood cells were studied, six were shown to have highly skewed X-inactivation, with more than 95% of cells expressing the normal *TAZ* allele - a pattern not seen in 148 normal female controls. Lesser degrees of skewing (65–95%) were present in another seven carriers [20] and the remaining three had a random pattern of X-inactivation (50–65%). Both highly skewed and non-skewed patterns of X-inactivation were present among females within the same family. It has been postulated that a post-inactivation selection mechanism might operate due to the TAZ protein causing respiratory chain abnormalities or other deleterious effects in multiple cell types [20].

#### Pathophysiology

*TAZ* encodes an acyltransferase that catalyses the remodeling of cardiolipin in mitochondrial membranes [6,21-23]. The fatty acyl chain configuration of cardiolipin is tissue-specific, with highly oxidative tissues having a cardiolipin with four linoleoyl moieties (tetralinoleoyl cardiolipin, “L4-CL”) as the predominant species. In mammalian cardiac and skeletal muscle L4-CL constitutes up to 70–80% of total cardiolipin [10,24]. *TAZ* mutations reduce formation of L4-CL in favor of cardiolipin molecules of different acyl composition and cause an accumulation of intermediate species carrying three rather than four linoleoyl acyl groups (monolysocardiolipins [MLCL]) [25,26]. This leads to a markedly increased MLCL:L4-CL ratio [27] and now forms the basis for a sensitive and apparently specific test for the disease [7,28].

**Figure 1:**
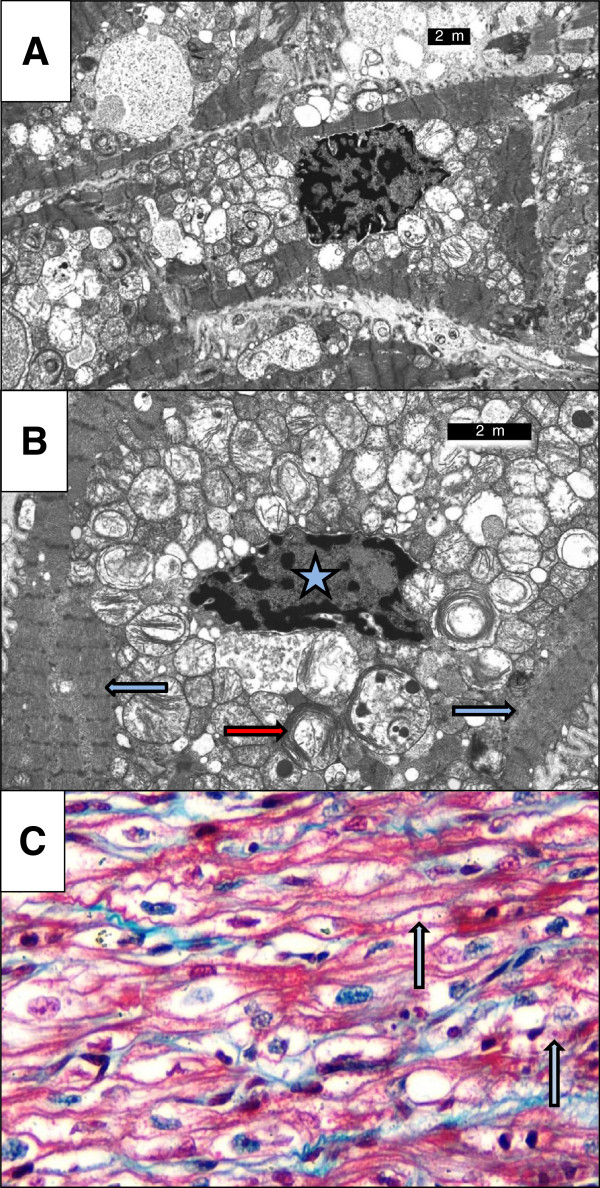
**Abnormal Mitochondrial Appearance in BTHS Dilated Cardiomyopathy. **(**A**) Electron micrograph of cardiac muscle biopsy from a patient with severe BTHS DCM. The myocyte cytoplasm contains increased numbers of mitochondria, most being larger than normal. Lipid is not increased but pools of non-membrane bound glycogen are evident. (**B**) Higher power electron micrograph. The mitochondria are enlarged and are crowded together, with many touching one another. The cristae are not parallel but are stacked, and many are in abnormal circular arrays (red arrow). The nucleus (starred) is centrally placed, but the myofilaments, with their cross-striations (blue arrows), are displaced to the periphery of the cell. (**C**) High power photomicrograph of myocardium stained with Masson-Trichrome stain. The myocytes are vacuolated with pale areas around the nucleus. In some giant mitochondria are visible as rounded, red bodies [arrows]. Such giant mitochondria are very suggestive of mitochondrial pathology and reflect the giant mitochondria seen on electron microscopy.

Cardiolipin has an important role in maintaining mitochondrial structure [29], associates with a number of mitochondrial proteins (reviewed in [30]) and is also involved in mitochondrial apoptosis (reviewed in [31]). In particular, cardiolipin stabilises highly ordered respiratory chain supercomplexes and optimises energy production in mitochondria [32,33]. Evidence for a role of cardiolipin in maintaining mitochondrial integrity is supported by varying degrees of structural and functional abnormalities of mitochondria isolated from BTHS patients [1,23,34,35]. An example of abnormal mitochondrial appearances from a patient with severe BTHS DCM is shown in Figure 1. 

Because research into the pathogenesis of BTHS in humans has until recently been hampered by the inability to make a mammalian model, most *in vivo* and *in vitro* studies have used yeast, fruit flies, zebrafish and cultured cells, as well as patient metabolic studies [36-42]. In 2010, an inducible *TAZ* knockdown mouse model of BTHS was developed using RNA interference technology [24,43]. *TAZ* knockdown for several months from birth produces a substantial reduction in the level of mature cardiolipin, an accumulation of MLCL species, and an increase of the MLCL/L4-CL ratio in cardiac and skeletal muscle. No significant cardiac effects were apparent in two-month-old mice, but by eight months of postnatal *TAZ* knockdown, a range of structural abnormalities of cardiac sarcomeres and mitochondria, and of cardiac structure and function, were apparent. The latter included left ventricular dilatation and dysfunction. The effect on skeletal muscle was more marked, with obvious ultrastructural changes apparent by two months. This model holds promise for furthering our understanding of the pathogenesis of BTHS and for testing candidate gene or drug therapies.

### Clinical description

The most widely recognised features of the disease comprise CM, skeletal myopathy, neutropenia, growth delay and increased urinary excretion of 3-MGCA [3]. However, a much broader phenotype is now recognised (Table 1). The majority of the information about BTHS comes from individual case reports and small cases series, although detailed analyses of 34 and 73 patients respectively were published in 2006 [44] and 2012 [4]. 

**Table 1:** Clinical features of Barth syndrome (common features are asterisked)

	
**Cardiovascular**	*Dilated Cardiomyopathy (DCM)
	*Left Ventricular Non-compaction (LVNC)
	*Prolonged corrected QT interval (QTc)
	Endocardial Fibroelastosis (EFE)
	Ventricular arrhythmia/Sudden cardiac death
	Undulating Cardiomyopathy
	Hypertrophic Cardiomyopathy (HCM) (rarely)
**Neuromuscular**	*Delayed motor milestones
	*Proximal myopathy
	*Abnormal fatigability
	*Exercise intolerance
**Neurological**	*Mild learning disabilities
	*Attention deficits
	Strokes (cardiac embolic)
**Haematological & Infectious**	*Neutropenia
	*Recurrent aphthous ulcers & sore gums
	*Perianal dermatitis
	Recurrent bacterial infections
	Septicaemia
**Endocrine & Metabolic**	*3-methylglutaconic aciduria
	*Constitutional growth delay with delayed bone age
	*Delayed puberty
	Hypocholesterolaemia
	Hypoglycaemia
	Lactic acidosis (often accompanies cardiac failure)
	Osteopenia
**Gastrointestinal**	*Oromotor feeding problems
	Episodic or chronic diarrhoea
**Dysmorphic features**	*Deep set eyes
	*Full cheeks
	*Prominent ears (older boys)
**Fetal**	Cardiomyopathy
	Fetal hydrops
	Male miscarriage and stillbirth

#### Cardiological aspects

CM is the major clinical feature in BTHS, with high prevalence in early life. Data from the BSF Registry show that 70% of BTHS patients are recognised to have CM in the first year (most of these presenting before six months of age) and that all those who developed CM did so by five years of age [4].

**Figure 2:**
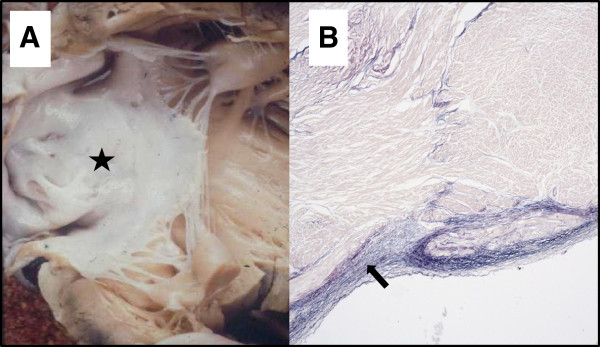
**Endocardial fibroelastosis in BTHS. **(**A**) The endocardium appears abnormally pale (starred) and (**B**) there is marked thickening (arrowed) of the endocardial surface on the corresponding photomicrograph (Van Gieson staining).

CM usually takes the form of DCM and can be accompanied by endocardial fibroelastosis (EFE, see Figure 2) [11]. 50% of patients have prominent left ventricular trabeculations, suggesting a form of LVNC. 

Hypertrophic CM (HCM) has also been described rarely [44,45], with both DCM and HCM being documented within a single family [44]. Left ventricular abnormalities may “remodel” with age; there may also be transition between relatively dilated and hypertrophic appearances, a feature described previously in children with LVNC and sometimes referred to as an “undulating” phenotype [46,47].

Unfortunately, there is often a significant delay in diagnosing BTHS in patients with CM [44,48]. A comprehensive Australian study [49] suggested that 4.8% of boys diagnosed with CM in 1987–96 had BTHS, and 7.2% of those with forms of cardiomyopathy other than hypertrophic. Similarly, data from the Pediatric Cardiomyopathy Registry of the USA suggests that 3–5% of all boys with CM will have BTHS and a relatively higher proportion of those with DCM and LVNC (personal communication, Dr Jeffrey Towbin). This substantial proportion suggests that all young males with unexplained DCM should be investigated for BTHS, especially those presenting as neonates or within the first year. The potential for fetal onset of CM in BTHS, which has been documented as early as 18 weeks gestation [50], has important implications which are discussed in the section on miscarriage and stillbirth.

Initial presentation of BTHS-associated CM may mimic viral myocarditis/CM or be precipitated by viral infection. For example, one patient presented acutely with severe DCM during primary infectious mononucleosis (personal communication, Dr Wilf Kelsall) and others coincident with a variety of proven respiratory viral infections. BTHS should therefore be included in the differential diagnosis of males presenting with DCM of apparent viral aetiology, especially where neutropenia is present (which could mistakenly be ascribed to secondary bone marrow suppression by viral infection). Stabilisation of CM, improved overall health, and steady growth often characterise the middle childhood years, leading parents to report a “honeymoon phase” in their boys. This tendency to spontaneous marked improvement of BTHS features may support clinicians in their belief that a patient in whom the diagnosis of BTHS has been missed is recovering from a strictly viral cardiomyopathy. Of note, although CM is a key feature of BTHS, not all BTHS patients have CM. Spencer et al. found three patients (10%, aged 3, 5 and 17 years), who had never had CM despite having affected relatives with clinically significant CM [44].

**Figure 3:**
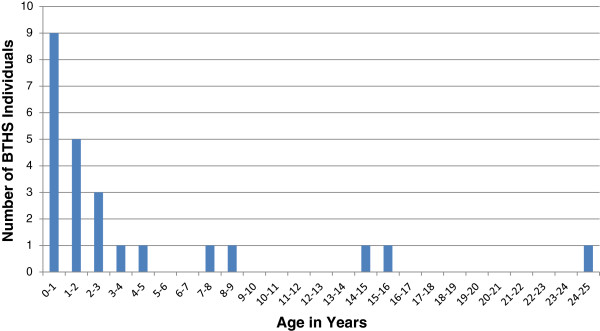
**Age at cardiac transplantation in BTHS patients. **Age is shown at the time of first transplant where multiple transplant procedures were required (data supplied by BSF). It should be noted that there were sometimes extended periods between initial listing for cardiac transplantation and performance of procedures (data unavailable).

There is a variable but usually overall good response to medical therapy for cardiac failure in BTHS. Spencer *et al*. [44] noted that more than 50% of patients normalised their ejection fraction and left ventricular diastolic volume Z Scores, although most needed to be maintained on standard cardiac medications for DCM throughout childhood and into the adult years. Thus, 16 of 30 fully evaluable BTHS patients in this study had normal left ventricular ejection fractions, although 10 of these were taking at least one cardiac medication. Some patients respond well to therapy initially but deteriorate after months or years of stability, necessitating cardiac transplantation [4]. Age at time of cardiac transplant is shown in Figure 3. 

There is also a risk of ventricular arrhythmia and sudden cardiac death in BTHS, which appears to be independent of the degree of CM [11,51,52]. These arrhythmias can occur at times of apparent good health. When studying a larger cohort, Spencer *et al*. found that nine out of 70 (13%) patients had a documented ventricular arrhythmia necessitating an implantable cardioverter defibrillator (ICD) [4]. Most of the documented serious arrhythmias have been in older children but life threatening arrhythmias have been observed in younger children, including two deaths related to ventricular arrhythmia in babies (unpublished observations). BTHS can also present as sudden cardiac death within families, as illustrated by a previous family history of sudden cardiac death in two BTHS patients with documented arrhythmias [53]. Spencer *et al* reported prolonged or borderline prolonged QTc in a high proportion of BTHS patients (43%) although this did not appear to correlate with episodes of documented ventricular arrhythmia [44]. Prolonged or borderline prolonged long QTc has been found in children with HCM and DCM due to other causes and may reflect the underlying cardiac muscle abnormality, which in BTHS includes myofibrillar disorganization [54]. Others have postulated that cardiolipin abnormalities could impede cross-communication between the endoplasmic reticulum and mitochondria, thus affecting calcium handling in cardiomyocytes and cardiac conducting cells [43].

#### Neutropenia

**Figure 4:**
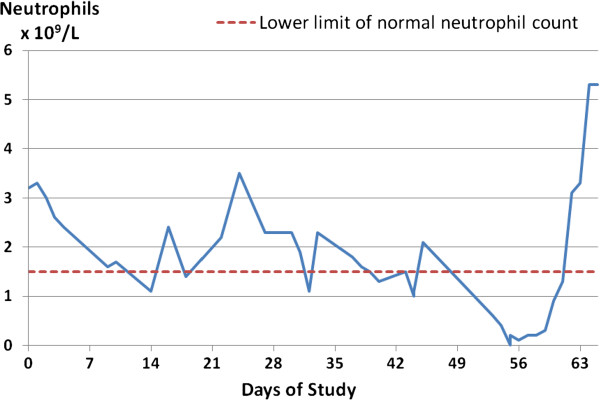
**Unpredictable neutropenia in a BTHS patient. **This graph shows a routine neutrophil profile from a BTHS patient who was not receiving G-CSF and had no clinical explanation for changes in neutrophil counts. It demonstrates that some BTHS patients can transition rapidly from severe neutropenia to high normal neutrophil counts. This emphasises the importance of not using presence/absence of neutropenia at presentation of male DCM as a critical determinant of whether testing for BTHS is performed. Furthermore, BTHS patients can present with bacterial infection in the context of a normal neutrophil count, infection having commenced when severely neutropenic just one or two days previously.

Neutropenia has been detected as a persistent or intermittent feature in 90% of BTHS patients, but completely normal neutrophil counts have been present over prolonged periods of follow-up in the remainder. It is important to note that 30% of cases are not neutropenic at initial presentation (Steward *et al*, manuscript in preparation). Neutropenia sometimes precedes other features, and has even been documented in a cord blood sample [1]. The neutropenia of BTHS takes many forms [9]; it can be severe and chronic (with many readings <0.5 × 10^9^/L) or truly cyclical, but most often is intermittent and unpredictable (example shown in Figure 4). Nadirs in the neutrophil count are associated with bacterial infection ranging from prolonged upper respiratory tract infections, mouth ulcers, inflamed gums and perianal dermatitis to overwhelming sepsis and multi-organ failure. Recognition of neutropenia and, therefore, correct diagnosis of BTHS can be delayed because neutrophil counts often increase to normal or supranormal within a few days of developing an infection [3]. 

Concurrence of severe neutropenia, CM, mitochondrial dysfunction, low muscle mass and a propensity to hypoglycaemia and lactic acidosis may all increase the likelihood of death in response to severe bacterial infection, making BTHS an underlying diagnosis to consider in patients dying with bacterial sepsis. Indeed, almost half of patients with a documented cause of death in Barth’s original paper (in a single, extended family) died from infectious rather than cardiac complications [1].

**Figure 5:**
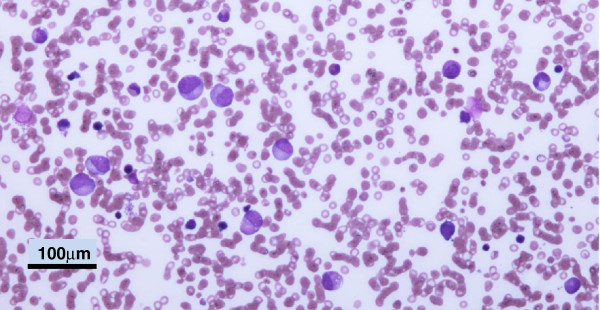
**Bone Marrow Appearances in BTHS. **A bone marrow aspirate from a patient with BTHS showing typical myelocyte arrest. There is an excess of monocytes and absence of mature neutrophils.

Neutropenia in BTHS is associated with myelocyte arrest on bone marrow aspiration [1] (see Figure 5) and compensatory monocytosis peripherally [3]. Although absolute neutrophil counts (ANCs) for some boys fluctuate within a relatively narrow range, others have wide and unpredictable swings in their ANCs. This complicates treatment with granulocyte colony stimulating factor (G-CSF), which is widely used in the management of neutropenic patients (the BSF Registry reports use of G-CSF in half of their registered cohort of BTHS patients [4]). 

The mechanism of neutropenia and infection in BTHS is under continuing debate. Barth *et al* showed in their original studies that neutrophil function was normal [1]. It has since been postulated that there may be increased neutrophil apoptosis or increased clearance of neutrophils by tissue macrophages, although this has not been supported in studies [55,56]. This has led to the hypothesis that the defect in BTHS might operate at the level of neutrophil precursors in bone marrow; Aprikyan *et al* transfected *TAZ*-specific shRNA constructs into human myeloid progenitor HL60 cells and showed that knockdown of *TAZ* gene expression by several constructs was associated with elevated dissipation of mitochondrial membrane potential, compared to that of control cells transfected with scrambled shRNA [57].

#### Neurological aspects & skeletal myopathy

Skeletal myopathy is widely recognised in BTHS, and most boys have at least mildly delayed gross motor milestones [44,58]. Muscle weakness is predominantly proximal and non-progressive during childhood, and Gower’s sign may be present. Grip strength can be reduced, tending to improve through the first decade and stabilise during adolescence, although distal muscle weakness is not a prominent component of the disease [44]. Patients with BTHS are able to walk but often find normal activities, such as kicking a ball or running, difficult. The evolution of disabling myopathy has been described at between 43 and 50 years in one man subsequently diagnosed with BTHS; his electromyogram was consistent with severe, chronic myopathy and creatine phosphokinase was slightly elevated [17].

Boys tend not to have classic myopathic facies, or involvement of extra-ocular or diaphragmatic muscles. Poor tone can result in lumbar lordosis. The typical marked reduction in muscle bulk, and therefore much lower than expected weight-for-height, can result in misdiagnosis as “failure to thrive”. Easy fatigability is a major problem in BTHS and some boys use mobility aids to conserve energy. Limitations of functional capacity are marked and likely multifactorial in nature, including limited cardiac reserve and diminished extraction/utilisation of oxygen by skeletal muscles [59].

Screening patients with unexplained mitochondrial myopathy has already identified one BTHS patient [28]. Diagnostic investigations for BTHS should therefore be considered in any boy with idiopathic myopathy or “mitochondrial disease” where no cause can be found on routine screening of relevant genes. Investigation of myopathy by invasive muscle biopsy has yielded a variety of results including increased fat vacuolisation/atrophy of type I fibres and increased subsarcolemmal spaces, normal or abnormal mitochondria, and variable changes in respiratory chain complexes [1,11,17,60-62]. It is, however, important to note that muscle biopsy has no role in the elective diagnosis of a patient with suspected BTHS.

BSF Registry self-reported data indicates that 48% of boys aged 7 years or older had some form of learning disability and that 33% required some form of special education [4]. A small study demonstrated significantly weaker visuospatial and visual motor scores on neuropsychological testing, and a non-significant tendency towards lower scores in mathematics, than in a control population [63]. Detailed neuropsychological assessment of a larger patient group is currently being performed (personal communication, Dr. Vanessa Garratt).

Stroke is also a significant risk in BTHS patients, mostly in the context of severe cardiac failure and possibly related to clot formation in the increased ventricular trabeculations often seen in BTHS. Middle cerebral artery occlusion has been reported in one 18-year-old patient with severe DCM [64] but 12 other patients are known to the BSF, including one who developed fatal clots shortly after going onto a Berlin Ventricular Assist Device (unpublished observations, Valerie Bowen).

#### Metabolic aspects

Many boys show a five to 20-fold increased level of 3-MGCA on quantitative analysis of urinary organic acids and BTHS is therefore also known as ‘type II 3-methylglutaconic aciduria’. 3-methylglutaric acid and 2-ethyl-hydracrylic acid levels are also increased [3]. However, cases have been reported where urinary 3-MGCA levels have been normal in patients with *TAZ* mutations [45,50,61,65-67] and levels may vary considerably even within a 24 hour period [9]. 3-MGCA is also elevated in a collection of disorders of widely varying phenotype (reviewed in [68]), making this a test with poor diagnostic specificity.

Although many boys with BTHS never experience significant metabolic problems, a range of metabolic abnormalities have been reported which may alert clinicians to consideration of the diagnosis. These include low pre-albumin levels (79%), decreased low density lipoprotein cholesterol (56%), hypocholesterolaemia (24%), mildly elevated creatine kinase (15%), lactic acidosis, hypoglycaemia, reduced plasma carnitine levels, raised serum transaminases and mild hyperammonaemia ( [3,44,69]; incidence figures taken from reference [44]). Lactic acidosis and hypoglycaemia appear to be more common in the neonatal period and infancy. Several cases of acute metabolic decompensation and death have been described in neonates [52,69].

Ongoing studies on the intermediary metabolism of BTHS suggest that secondary abnormalities of citric acid cycle function sufficiently affect anaplerosis and amino acid metabolism to cause inadequate muscle protein synthesis (unpublished observations, Dr Richard Kelley). Various attempts at targeting the metabolic derangements in BTHS have been tried, including the use of L-carnitine [70] or pantothenic acid [71]. Whilst some patients with BTHS are able to tolerate pharmacologic doses (50–100 mg/kg/d) of L-carnitine, there is no evidence that these amounts are therapeutic and carnitine is therefore not usually given unless total plasma carnitine levels are low. Most patients treated with pantothenic acid supplements have failed to show benefit (representative examples reported in [72]). More recently, the identification of consistently low levels of specific amino acids have led to dietary supplementation with arginine and other amino acids but there is as yet no published data on these observations or treatment.

#### Growth delay

**Figure 6:**
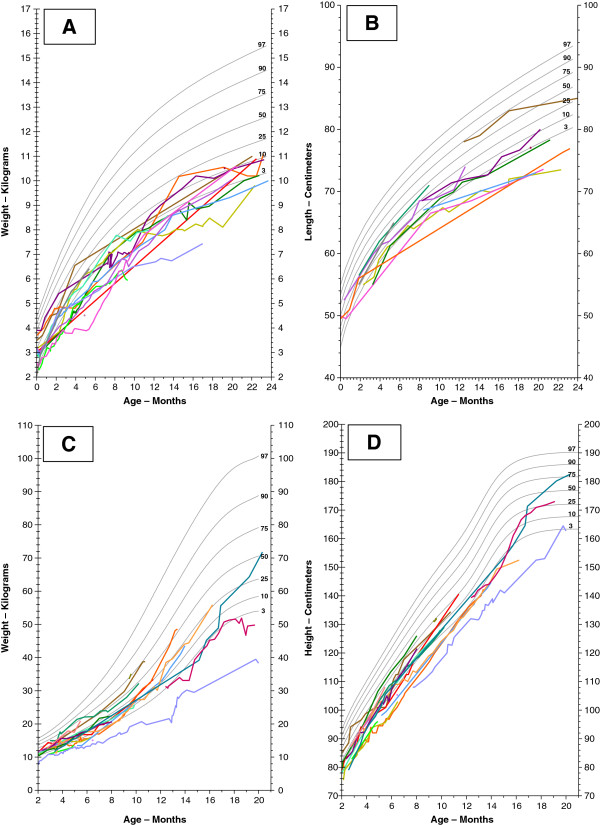
**Longitudinal growth data. **Growth data from 23 patients attending the NSSBSS clinics, plotted as weight and length respectively in the first two years (**A** and **B**) and weight and height from 2–21 years (**C** and **D**). One patient followed to 20 years underwent cardiac transplantation at 20 months and another one followed to 21 years has received both cardiac and renal transplants (at 2 and 15 years respectively). The three oldest patients illustrated in (**D**) demonstrate constitutional growth delay with continued growth to as late as 21 years.

**Figure 7:**
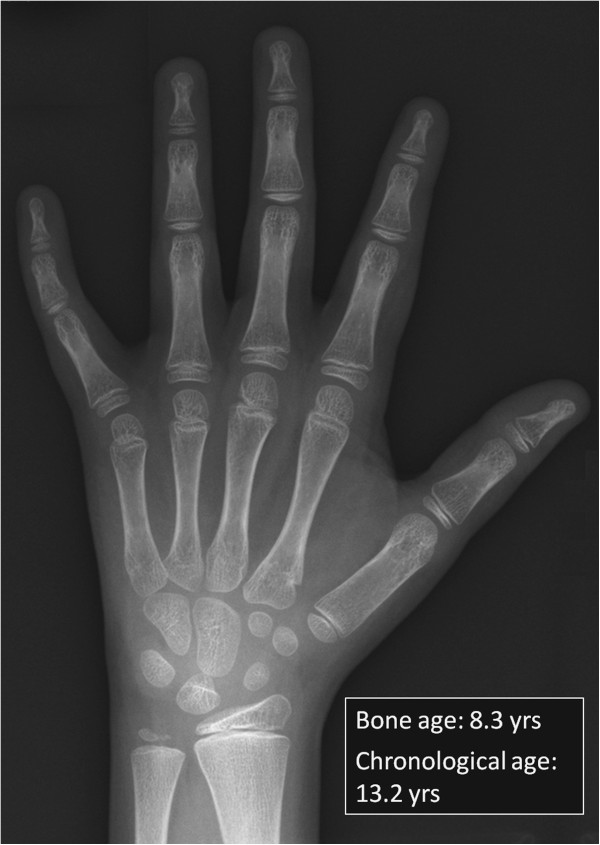
**Delayed Bone Age. **This patient showed severe constitutional growth delay with a bone age of 8.3 years on the Tanner and Whitehouse scale at a chronological age of 13.2 years.

Growth delay is seen in both BTHS patients [4] and cellular models of the condition (reviewed in [73]). Hypothetically, many factors could contribute to growth delay, including poor nutrition, diarrhoea, CM, sore mouth/gums and recurrent infection. However, to a considerable extent the growth pattern of BTHS conforms to that of typical constitutional growth delay with delayed puberty. Longitudinal growth data of a representative population (patients attending the NSSBSS clinics) is shown in Figure 6. Delayed bone age has been present in all boys attending this clinic, ranging between 8 months and 2 years 6 months, although more extreme delay has been noted (as exemplified by the boy whose wrist X-ray is shown in Figure 7). 

Spencer *et al*[44] found that 58% of patients less than 18 yrs were below the 5th centile for weight and at or below the 5th centile for height. By contrast, those older than 18 years had a mean weight on the 13th percentile (range <1–63%) but mean height on the 50th percentile (range 8–90%). This change is effected by a delayed growth spurt at 15–21 years of age, which can take boys rapidly from a low growth centile to even above their midparental height in a few cases. This may be accompanied by worsening of cardiac function. The period of rapid pubertal growth often occurs without commensurate increase in food intake, with the result that many boys may appear to be relatively emaciated, and supplemental feeding via nasogastric or gastrostomy tubes has sometimes been given. Normalised growth curves for BTHS patients have been prepared from BSF Registry data [4].

A study of 22 BTHS patients aged between 4 months and 24 years reported subnormal levels of growth hormone (GH) below 14.4 years, but levels higher than controls above this age, most marked at 19–20 years [74]. Insulin-like growth factor-1 (IGF-I) deficiency has been reported; as expected for constitutional growth delay, the patient had normal GH release and no other sign of pituitary dysfunction. One additional BTHS patient with typical constitutional growth delay and low IGF-1 level was treated with GH for one year, but showed no change in growth velocity. However, two boys with BTHS who showed atypical growth arrest in their middle childhood years met standard criteria for GH deficiency and responded to GH treatment (unpublished observations, Dr Richard Kelley). This occurrence of two cases of *bona fide* central GH deficiency in such a small patient cohort suggests that there could be an increased risk for central GH deficiency in BTHS.

It should be noted that not all children display poor growth. Spencer *et al* documented that nearly 25% of boys younger than 18 years had weight above the 20th percentile and more than 50% had normal or increased BMI [44]. Boys with BTHS with BMI greater than the 25th centile have markedly abnormal truncal adiposity (unpublished observations, Nicol Clayton & Dr Richard Kelley).

#### Feeding problems

There is little published information about feeding problems in BTHS, but many boys are slow to transition to solid food, and have a degree of oromotor weakness, leading to a sensitive gag reflex, poor chewing skills, extended mealtimes and small portion sizes. When accompanied by slow growth along the lower centiles and concern about hypoglycaemia, this situation often leads to supplemental feeding. The BSF Registry reports that one third of BTHS patients have required a nasogastric or gastrostomy tube for feeding at some stage [4].

#### Miscarriage & stillbirth

The first case of BTHS detected prenatally was published in 1997 [75]. It is now known that CM due to genetically proven BTHS can develop as early as 18 weeks gestation [50]. A survey published in 2010 showed that one third of BTHS families in the UK have a history of excessive male fetal and neonatal losses, establishing that BTHS could lead to isolated or recurrent male fetal death [5]. In six families, nine babies were stillborn, 14 died in early life, and a further nine miscarriages were suspicious for BTHS. Importantly, there were no female losses from the second trimester through to term in these families, and one woman (since proven to be a BTHS carrier) lost all three of her male babies as second trimester miscarriages or stillbirth at term. Combined with evidence of CM *in utero* and age-related expression of *TAZ*, this provides support for a role of *TAZ* in embryonic, fetal and neonatal life [50,75].

Further evidence comes from zebrafish and mouse TAZ knockdown models. In zebrafish, studies showed that TAZ RNA was expressed at high levels in many tissues in the early stages of embryogenesis, becoming restricted to the heart in late gestation, and that TAZ expression was essential for normal development [39]. In the mouse model of BTHS, if females are fed doxycycline (resulting in gene knockdown) from the start of gestation throughout pregnancy, their offspring develop early cardiac diastolic dysfunction, hypertrabeculation and noncompaction [76]. BTHS therefore appears to be an important differential diagnosis of male fetal loss where there is evidence of hydrops and CM.

#### Dysmorphology

**Figure 8:**
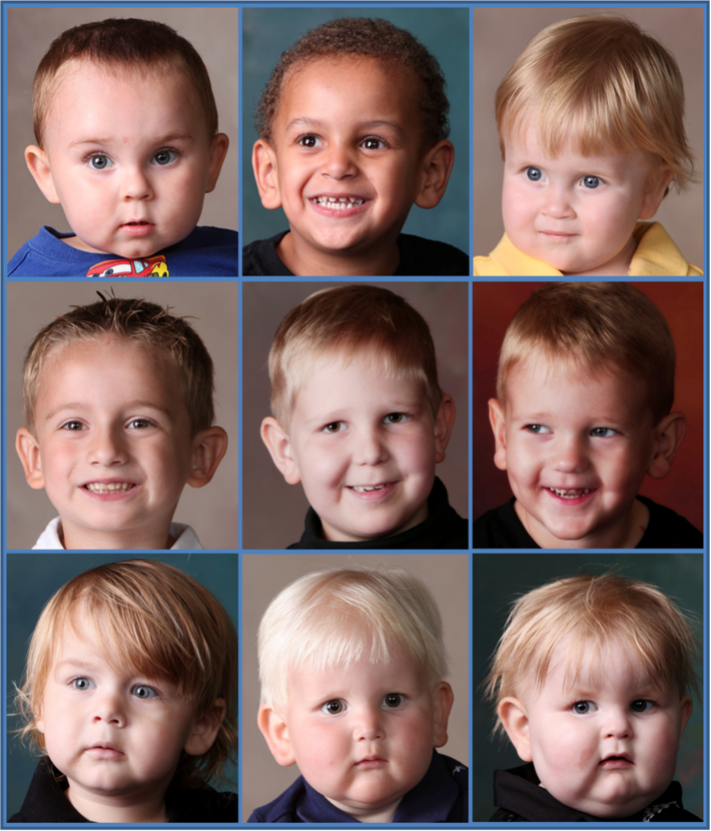
**Facial Appearance. **Young boys with BTHS have consistent facial features with a tall, broad forehead, round face with full cheeks, prominent ears and deep-set eyes. These features become less evident during puberty and into early adulthood, although the eyes may remain deep set and ears prominent [77].

Boys with BTHS show remarkable similarities in facial features, especially from infancy to early adolescence [77]. In early childhood, the face is round with full cheeks and an overall “cherubic” appearance (see Figure 8), whereas after the first decade and especially after puberty, the head and face become narrowed and the ears more prominent. During adolescence, there is development of gynoid proportions and, in some, relative truncal fat distribution. 

### Diagnosis and diagnostic methods

Historically, diagnosis of BTHS has principally relied on identifying boys with the combination of cardioskeletal myopathy, neutropenia and 3-MGCAciduria. However, reliance on this diagnostic triad has been fraught with problems. Firstly, one third of patients will not be neutropenic at presentation, and some boys will never display neutropenia. Secondly, the urine level of 3-MGCA may be normal or, being only mildly to moderately increased, is sometimes missed by laboratories that do not perform quantitative studies or are not sensitive to the significance of mild to moderate 3-MGCAciduria.

Such problems in diagnosis have now been partially overcome by the development of a straightforward diagnostic assay measuring the MLCL:L4-CL ratio [26,28,78], which can be applied to a variety of cells and tissues, including stored dried bloodspot cards [7]. This ratio has shown 100% diagnostic sensitivity and specificity for BTHS [7,28,78] but to date is only available in a few centres worldwide. Cardiolipin ratio analysis allows definitive diagnosis at a much lower cost than genetic testing, and could now be considered a routine component of the testing of males with idiopathic DCM, especially those presenting in the neonatal or infant period. Abnormal cardiolipin ratio testing can then be followed up with targeted genetic testing for *TAZ* mutations. In addition, if stored tissue (e.g. fibroblasts, dried bloodspots) is available, retrospective diagnosis can be attempted by cardiolipin ratio testing, *TAZ* analysis or a combination of the two. It is also important to note that most commercially available mutation screening panels for DCM and LVNC will include screening for a *TAZ* mutation (although this should be confirmed with the individual laboratory).

Clinical scenarios where exclusion of BTHS should be considered are summarised in Table 2 and the relevant diagnostic tests available are described in Table 3. 

**Table 2:** Clinical indications for exclusion of BTHS

**Recommendations for exclusion of BTHS**	**Clinical scenario**
**Consider routinely**	· Fetal Cardiomyopathy
	· Male children with DCM ± EFE, especially those with neonatal / infantile onset
	· X-linked cardiomyopathy of any type (DCM, LVNC, HCM)
	· Cardiomyopathy with LVNC or LV modeling defects
	· Cardiac arrest / cardiac arrhythmia with echocardiographic abnormality consistent with BTHS
**Consider with relevant accompanying features** e.g. X-linked family history, growth failure, feeding problems, delayed motor milestones, lethargy/easy fatigue:	· Multiple male miscarriages / stillbirths / unexplained deaths
	· Neonatal / infantile hypoglycaemia / lactic acidosis
	· Viral cardiomyopathy
	· Unexplained neutropenia, mitochondrial disease or proximal myopathy
	· Serious unexplained bacterial sepsis
	· Severe feeding problems, persistent episodes of vomiting / diarrhoea
	· Growth failure / delayed puberty / delayed bone age
	· Unexplained ventricular arrhythmia or prolonged QTc interval

**Table 3:** Diagnostic tests for BTHS

**Test**	**Description**
**Urinary 3-MGCA Testing**	Most cases of BTHS will be suggested by the finding of a 5-20 fold elevation of 3-MGC excretion on quantitative urinary organic acid analysis. As this test is non-specific, confirmation of the diagnosis is required by TAZ mutation/cardiolipin ratio testing.
	***N.B.****Multiple reports suggest that urinary 3-MGCA levels may be normal on single specimen testing in patients later shown to have BTHS*[45,50,61,65-67]*. A negative 3-MGCA test in a candidate patient must therefore be confirmed by cardiolipin ratio testing or TAZ sequencing in order to reliably exclude BTHS.*
**Monolysocardiolipin/Cardiolipin (MLCL:L4-CL) Ratio Testing (where available)**	This test offers the best combination of speed, cost and diagnostic accuracy, with 100% diagnostic sensitivity and specificity reported in several series, but is currently only available in a few laboratories [7,28,78].
	Testing can be performed on blood specimens (sent by routine postal services), stored tissue or dried blood spots.
**TAZ Gene Sequencing:**	TAZ gene sequencing can be targeted selectively to those with an abnormal cardiolipin ratio test or performed in all candidate patients where cardiolipin testing is not available. It is increasingly being offered as one of the genes sequenced simultaneously in sequencing panels for investigation of cardiomyopathy. It also has an important place in the investigation of patients/families where appropriate samples for biochemical testing are not available from suspected index patients.

### Antenatal diagnosis and genetic counselling

Clinical genetic assessment has a major role in assessing potential BTHS patients/families. BTHS should be strongly suspected where the parents are healthy but there is a history of DCM, early death or male miscarriage/stillbirth affecting male siblings or male antecedents related through the maternal line of the family.

Once the causative mutation has been identified, pedigree analysis, cascade testing and the offer of antenatal diagnosis to proven carriers should be given high priority. If the mother of an index patient is shown to be a carrier, this will concentrate on female relatives in her family line and brothers with a suspicious history of ill health (this may have caused problems in early childhood but subsequently improved). All the daughters of a BTHS male would be expected to be obligate carriers but none of his sons.

Despite an isolated report of prenatally detected BTHS [75], Bleyl et al. showed that fetal ultrasound was not reliable in predicting LVNC [79] and indeed there is no clear evidence that fetal ultrasound screening and early delivery of affected fetuses can improve outcome. In BTHS families, molecular genetic analysis using DNA from chorionic villus sampling and/or amniocentesis preceded by fetal sexing using free fetal DNA analysis on maternal blood to identify male pregnancies will allow definitive antenatal genetic diagnosis [80,81].

### Differential diagnosis

The huge variety of clinical features attributed to BTHS means that the differential diagnosis is potentially very wide. This is especially true for those boys with a partial phenotype who present with only one or a few clinical features of BTHS. However, key differential diagnoses include: 

• Hereditary cardiomyopathy (including autosomal dominant, autosomal recessive, X-linked and mitochondrial forms)

• Dilated cardiomyopathy of endocrine or metabolic aetiology. The most important differential diagnosis is DCM with ataxia (DCMA) syndrome, an autosomal recessive BTHS-like disorder first described in the Canadian Dariusleut Hutterite population in 2006 [82] and more recently diagnosed in a pair of Finnish brothers [83]. This is caused by homozygous mutations in the *DNAJC19* gene which encodes a protein previously localised to mitochondria in cardiac myocytes and thought to play a role in mitochondrial matrix protein import [84]. Males and females are affected, with a high incidence of early onset DCM plus noncompaction cardiomyopathy with prolonged QTc interval, skeletal myopathy, microcytic anaemia, non-progressive cerebellar ataxia, testicular dysgenesis, growth failure and 3-MGCA (termed type V methylglutaconic aciduria) and 3-methylglutaric aciduria. Neutropenia has not been described.

• Nutritional cardiomyopathy (including thiamine, selenium, carnitine and vitamin D deficiency)

• Idiopathic mitochondrial disease

• Cyclic or idiopathic neutropenia

### Management including treatment

Standard heart failure management has included the use of angiotensin converting enzyme inhibitors, beta blockers, digoxin and diuretics, although there are no published studies analyzing the efficacy of these. Many BTHS patients appear to respond to this conventional medical therapy and no specific cardiac medications have been shown to be of particular value or risk. Long term surveillance for arrhythmias is recommended in all patients. The finding of ventricular arrhythmia or symptoms such as syncope should prompt additional testing and consideration of placement of an ICD.

14% of patients in the BSF Registry have required cardiac transplantation [85]. This procedure has generally shown good results despite the high pre-operative risk [48], although there is one reported case of an unusual non-Epstein-Barr virus-associated T-cell lymphoma after cardiac transplantation which resulted in the death of the patient due to intolerance of chemotherapy [86]. The Berlin EXCOR left ventricular assist device has been used to bridge the gap until a donor heart can be sourced in some boys with severe cardiac dysfunction [47]. Major risks include stroke due to clots forming within the chambers and infection aggravated by neutropenia.

Most patients, even those with fully adequate nutrition, will show a decreasing growth velocity during their first two years. Provided that patients with BTHS grow along or parallel to a low percentile after the third year, their diets should not be augmented with the aim of driving them back towards their birth weight and length percentiles, since this often produces vomiting, can cause longstanding food aversion and may contribute to development of truncal obesity. Cornstarch supplements may be used at bedtime (as in patients with glycogen storage disease) to provide an alternative source of glucose production and so limit the degree of muscle wasting resulting from overnight fasting [87].

Patients with symptomatic neutropenia are usually treated with a combination of subcutaneous G-CSF and prophylactic antibiotics. G-CSF is typically commenced at a dose of 2–3 μg/kg/dose at a frequency varying between twice weekly and alternate daily according to the severity of their neutropenia, allied infections and drug responses (Dr Colin Steward, manuscript in preparation). The aim is to raise the average of the neutrophil count rather than to permanently normalise it – since trying to achieve the latter can lead to major neutrophilia due to superimposition of G-CSF effects on the highly variable neutrophil count typical of BTHS patients. Many patients will show marked symptomatic improvements (prevention of aphthous ulcers and sore gums, reductions in bacterial infections and sometimes reduced lethargy). Prophylactic antibiotics are frequently used to reduce the risk of serious infections, especially in boys who are intermittently neutropenic but not receiving G-CSF.

No drug or food supplement has so far been shown to be conclusively beneficial. The value of pantothenic acid [71] has not been proven and single reported patients have worsened soon after introduction of L-carnitine supplements or deteriorated whilst on supplementation [71,72].

In addition to medical and surgical intervention, many other specialists play a role in the management of BTHS including physiotherapists and occupational therapists, speech and language therapists, psychologists and educational support workers. Patients with this complex disease are therefore best managed by a multidisciplinary team approach within specialised clinics.

### Prognosis

Historically, most boys with BTHS died during fetal life through to infancy from either heart failure or overwhelming infection. A paper published in 2005 showed that 70% of retrospectively-diagnosed brothers of BTHS patients had died before the diagnosis was established in the family. This contrasted with patients identified prospectively and managed proactively, for whom mortality had fallen to just 10% [88], emphasising the importance of early diagnosis. Despite this emphasis on early diagnosis, data from the BSF Registry shows a mean lag time of 3.3 years between first presentation and diagnosis [85].

**Figure 9:**
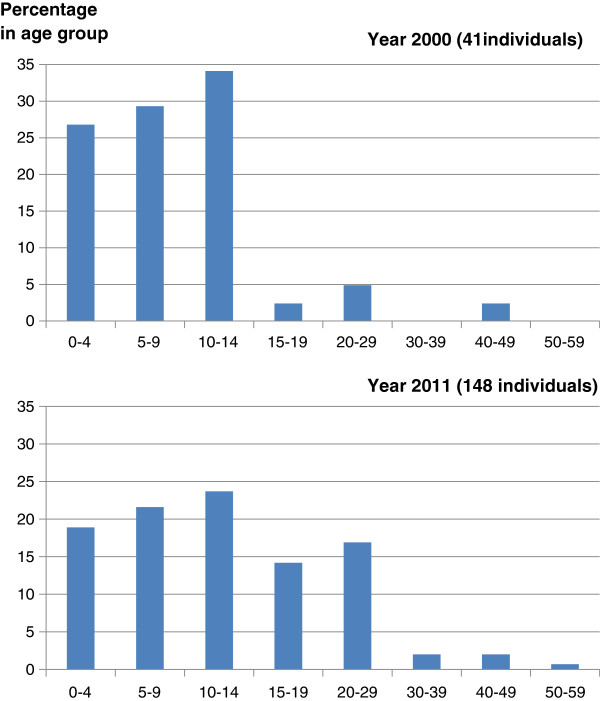
**Age distribution of BTHS patients. **The age distribution of living BTHS cases known to the BSF in 2000 (**A**) and 2011 (**B**)**.**

The BSF have collated data on the age of known BTHS patients (see Figure 9). In the year of its inception (2000), BSF knew of 41 living males with BTHS, of whom only 4/41 (10%) were over 15 years of age. By 2011 this had climbed to 53/148 (36%, data courtesy of BSF). Although likely to be subject to a degree of ascertainment bias, these figures suggest that males diagnosed with BTHS are surviving longer in response to improvements in supportive care. The oldest reported patient with BTHS is currently in his 50’s [75]. 

### Unresolved questions

There have been many major advances in our understanding of BTHS, especially in terms of aetiology, the breadth of the disease phenotype and diagnostic methods. However many unanswered questions remain in areas such as the variability of clinical severity with age in individual patients or between family members, lack of phenotype/genotype correlation and the exact mechanism by which altered cardiolipin metabolism causes BTHS. This has hindered understanding of the unusual features of BTHS (such as the waxing and waning of neutropenia, fluctuating severity of cardiomyopathy and constitutional growth delay), as well as the approach to managing patients. There is also no definitive epidemiological data on the frequency of BTHS.

## Conclusions

For 25 years after its first description, BTHS was generally regarded as an extremely rare disease. It was usually only considered when patients presented with a combination of CM, neutropenia and excessive urinary excretion of 3-MCGA. We have described how this only outlines a partial view of the disease, which affects many systems from fetal through to adult life. We recommend that investigation for BTHS should now be regarded as essential in male neonates, babies and young boys presenting with idiopathic DCM or LVNC, and in males with unexplained ventricular arrhythmia or sudden death. It should also be considered in the following scenarios: boys with idiopathic neutropenia (particularly where this is intermittent and unpredictable); those with unexplained severe bacterial sepsis accompanied by features such as growth delay, unexplained failure to thrive or motor delay; in boys with persistent unexplained feeding problems; “idiopathic” mitochondrial disease with myopathy and miscarriage/stillbirth where it is clear that either an X-linked pattern of inheritance and/or CM is present. Early identification allows wider family screening and antenatal counselling, as well as allowing disease appropriate management, which can radically reduce early mortality.

## Consent

Written informed consent was obtained from the patient's guardian/parent/next of kin for publication of this report and any accompanying images.

## Abbreviations

3-MGCA: 3-methylglutaconic acid; ANC: Absolute neutrophil counts; BSF: Barth Syndrome Foundation; BTHS: Barth syndrome; CM: Cardiomyopathy; DCM: Dilated cardiomyopathy; EFE: Endocardial fibroelastosis; EFE2: Endocardial fibroelastosis type 2; G-CSF: Granulocyte colony stimulating factor; HCM: Hypertrophic cardiomyopathy; ICD: Implantable cardioverter defibrillator; IGF-1: Insulin-like growth factor-1; L4-CL: Tetralinoleoyl cardiolipin; LVNC: Left ventricular non-compaction; MGA2: 3-methylglutaconic aciduria type 2; MLCL: Monolysocardiolipin; *TAZ*: Tafazzin; VF: Ventricular fibrillation; VT: Ventricular tachycardia.

## Competing interests

The Barth Syndrome Foundation (http://www.barthsyndrome.org) and Barth Syndrome Trust (http://www.barthsyndrome.org.uk) have funded the processing charge for this article. The authors declare that they have no competing interests.

## Authors’ contributions

SC performed the literature search and drafted the initial manuscript. CGS conceived and supervised the project and critically revised the manuscript. All other authors were involved in the conception and writing of the manuscript, and have read and approved the final manuscript.
